# Supplementary figures and images for: Characteristics of Neurokinin-3 Receptor and Its Binding Sites by Mutational Analysis
Source: Biology (Basel). 2021 Sep 27;10(10):968. doi: 10.3390/biology10100968 (PMC8533089; doi:10.3390/biology10100968)

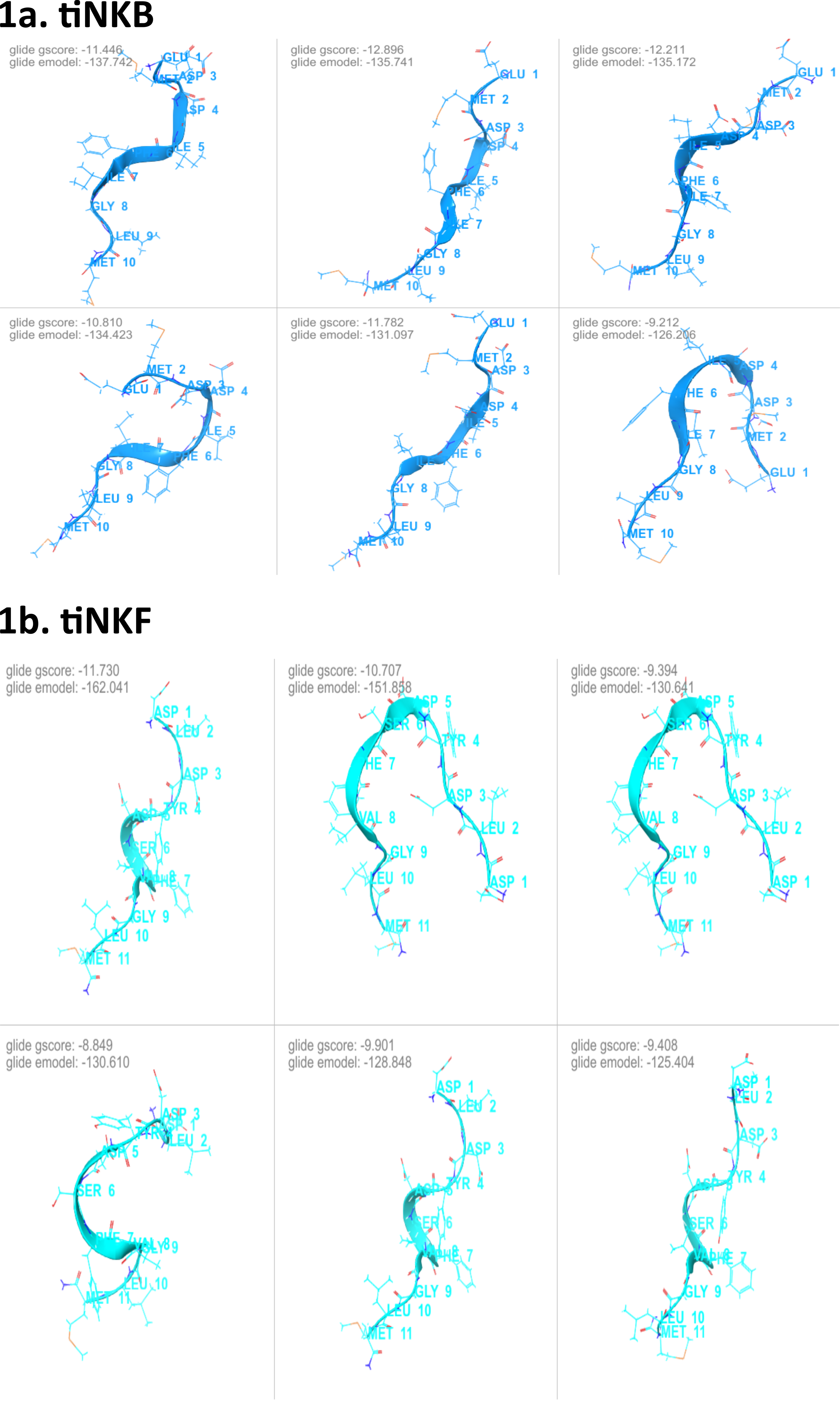

Supplement: Supplementary file 1 [file biology-10-00968-s001.zip › fig S1.tif]

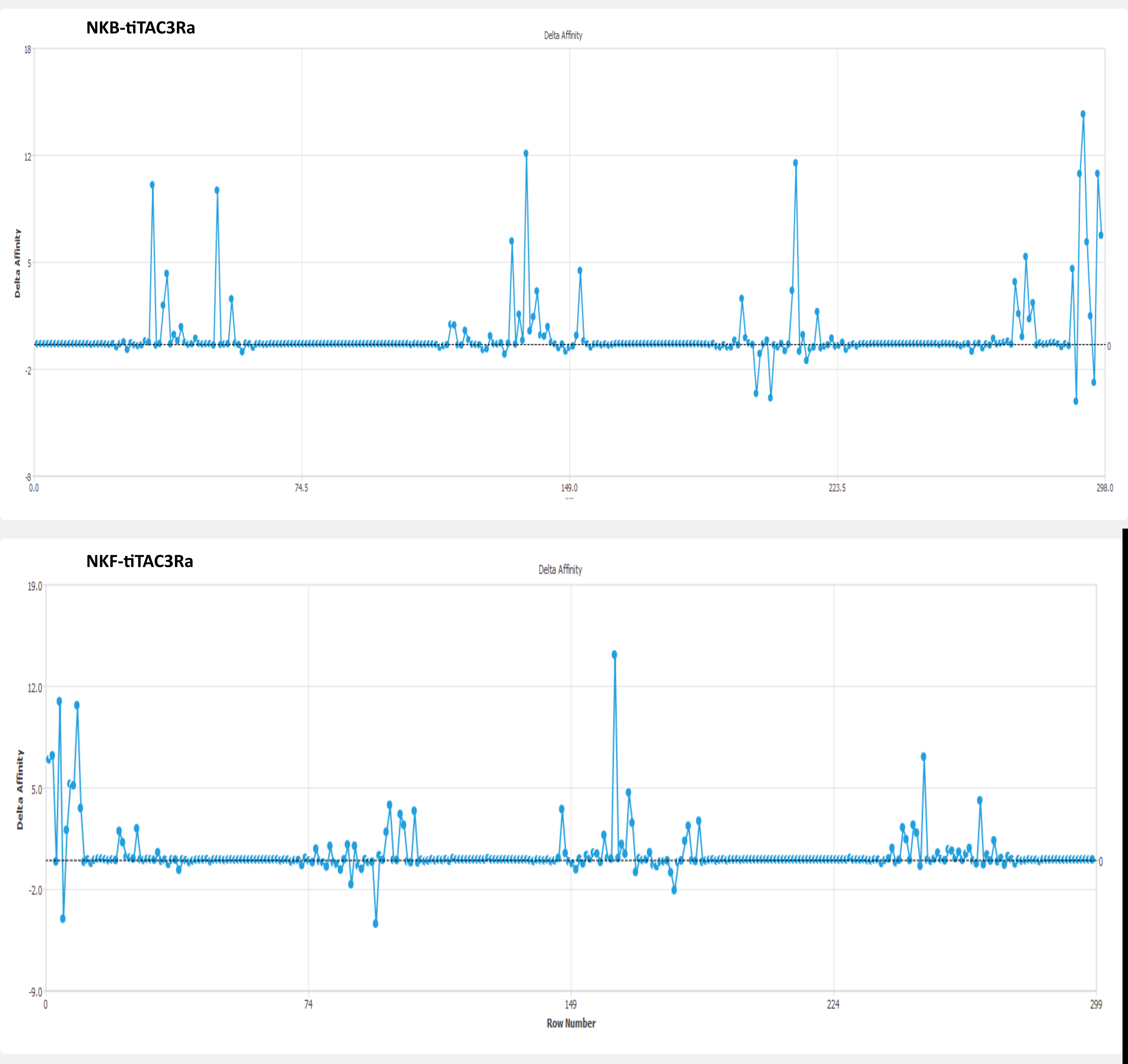

Supplement: Supplementary file 1 [file biology-10-00968-s001.zip › Fig.S2.tif]

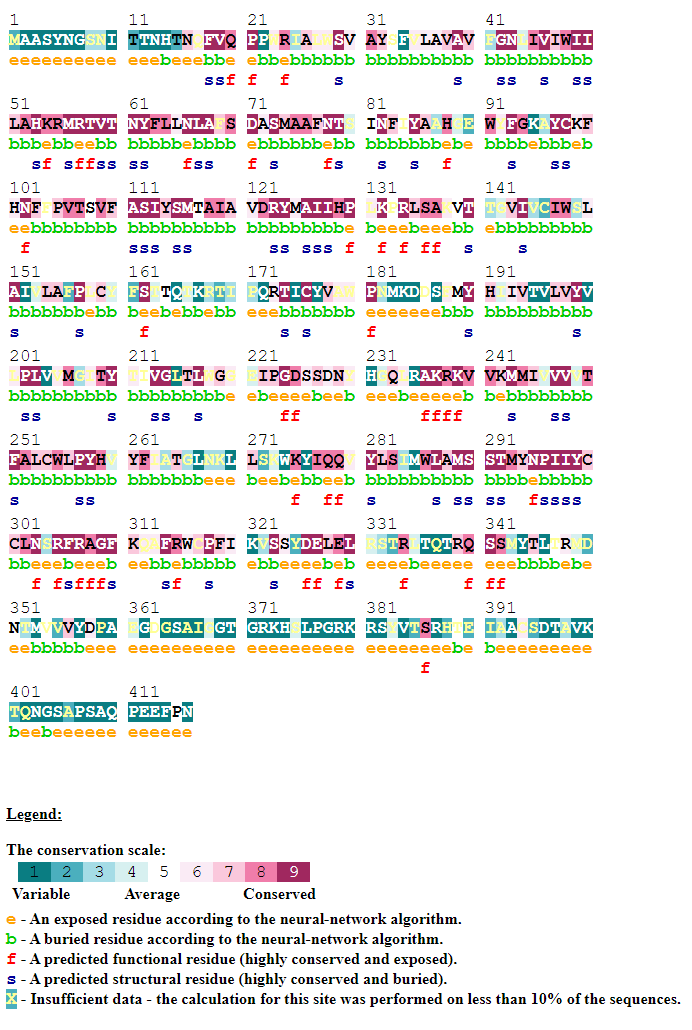

Supplement: Supplementary file 1 [file biology-10-00968-s001.zip › Fig.S3.tif]

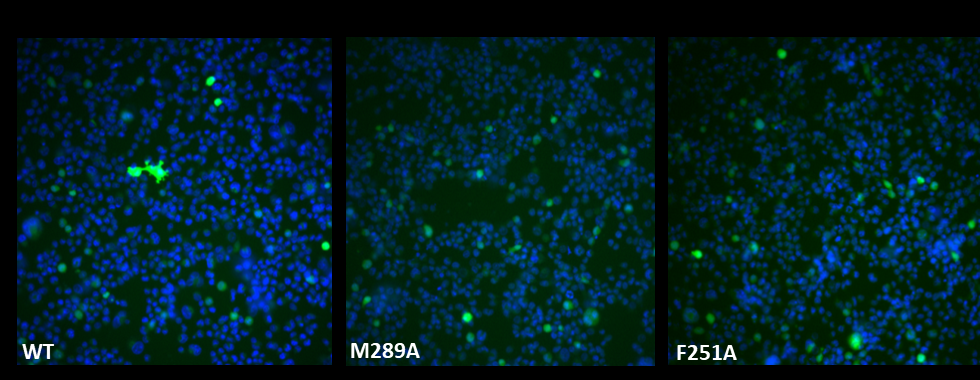

Supplement: Supplementary file 1 [file biology-10-00968-s001.zip › Fig.S4.tif]

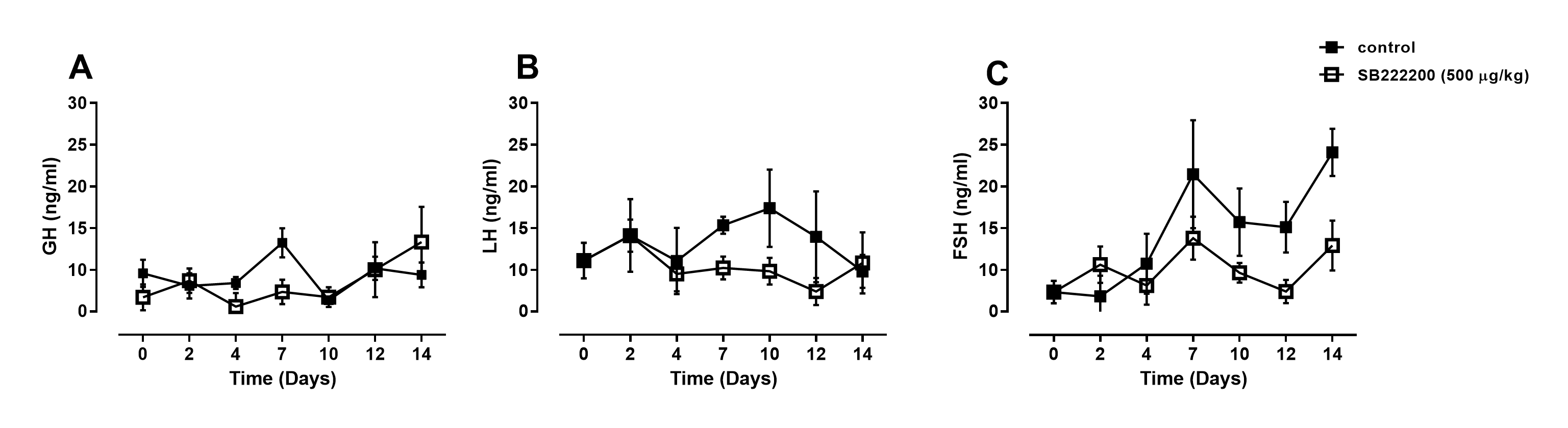

Supplement: Supplementary file 1 [file biology-10-00968-s001.zip › Fig.S5.tif]
